# Quo vadis palliative care in Africa?

**DOI:** 10.3332/ecancer.2013.ed29

**Published:** 2013-11-19

**Authors:** Anne Merriman

**Affiliations:** Founder of Hospice Africa, Director of Policy and International Programmes, Honorary Professor of Palliative Care at Makerere University and the Institute of Palliative Care in Africa at Hospice Africa Uganda

Many people, and especially governments, are still blind to the severe agonies that their populations suffer due to cancer.

## Introduction

In 1992, Hospice Africa was founded in Liverpool, UK with a vision to support cancer patients in Africa through affordable methods for the control of pain and suffering. In 1993, following a feasibility study in four countries, Uganda was chosen to roll out the initiative. Today, Hospice Africa Uganda is operating with a public health approach by training, up to degree level, health professionals who in their own countries are carrying forward palliative care to all those in need in Africa. Although palliative care provision has progressed greatly in recent years, there is still a need for all health professionals to play a greater part in relieving the suffering of cancer patients in Africa today.

## Setting the scene

Cancer treatment is rare for those in Africa. Radiotherapy is available in only 1/3 of countries and oncology drugs are often unaffordable. Populations and life expectancy, together with cancer incidence, are increasing. This, combined with late presentations, due to the fact that cancer is viewed as a death sentence, as many people witness relatives going to hospital only to come home in a box, means that 95% of cancer cases are incurable, except for the few more wealthy patients who can go abroad.

Considering that in most African countries more than 50% of cancer patients never see a health worker, many suffer gross disfigurement, pain and abandonment until death. The time has come for this suffering, brought about more by omission than intent, to be managed.

## The situation today in Africa for relief for those suffering from cancer

We in Africa today are in a similar situation as in 1986 [1], when the WHO first sent an appeal to all governments in the world, pointing out the discrepancies in availability of treatments and cures for cancer between the rich and the poor countries, resulting in tremendous suffering for patients and families. They pointed out that the only affordable and humanitarian approach was palliative care. Now that we have the knowledge of pain control and awareness of the analgesic ladder since 1986, as long as it is affordable, countries should not leave patients in pain just because a curable therapy is not available. Today, even with lots of success stories and advocacy, suffering akin to torture is still neglected by Governments, health professionals and even oncologists.

In 42 of the 57 African countries, the governments are still not willing to import and provide affordable morphine, which the WHO has recommended for severe pain, for their cancer patients. This was originally due to the high cost of manufactured oral morphine, however this can now be produced in an affordable manner close to the patient, without the “middle” man and abiding by all universal regulations (INCB). The main problem is still largely due to the myths and fears of misinformed governments, that those prescribing or giving morphine are addicts or supporting addicts. To ensure adequate pain relief for cancer patients, governments need to make affordable morphine freely available to patients in need. As this would need to be prescribed by a registered prescriber, increasing the number and training of prescribers is essential as well as the introduction of guidelines to ensure that the regulations of the Geneva conventions on controlled drugs are followed. The Ugandan Government has introduced these measures and is today seen as a model for Africa. Governments will be judged by their care for the most vulnerable of their populations; preventable pain, often akin to torture, is still commonplace in many African countries.

Yes, we health professionals must take some of the blame for the great suffering throughout Africa. We need to work together and to recognise the contribution that each can make to the comfort of patients and families at this special time of life. This is a plea for oncologists to work together with palliative care throughout Africa, supporting and using their knowledge about medications to relieve suffering ensuring that “nothing more can be done” never occurs. We need oncologists to join with the voices from palliative care groups, to advocate governments to allow the importation of powdered morphine so that affordable liquid morphine is available wherever a patient requires it (usually in the home [2]).

Africa is poor and hence cannot support the multiplication of specialties. Psycho oncology is part of palliative care and cannot stand alone otherwise it may fragment coordinated services for the underprivileged in resource-poor countries. At AORTIC it is in a separate stream. Why are they not put together?

As Palliative care (in Africa) uses generic medications to keep the cost down as much as possible the drug firms are not very forthcoming in funding our events or our advocacy. This means that we cannot always bring our best advocates in Africa to conferences. However combining psycho oncology with palliative care at the AORTIC conference would show, at least, that we are working together and this may make resources more available. Africa needs African experience to see how it is working. Palliative care as used in Western countries is not the same, culturally or economically. Let us be proud to be African but only if we recognise and meet the realistic needs of our patients today.

We also need to train health professionals in palliative care. Our degrees and training in oncology and radiotherapy, must include modules in palliative care. Teams providing oncology services need training in palliative care whereas degrees and training in palliative care need to include modules and sections on oncology. Our oncology nurses need to be dual trained so that they too can bring comfort to their patients. In this way we can move forward together and our patients and families can be at rest knowing that the service will refer patients for pain medication when necessary. We need to give the patient the chance to make decisions with choices regarding end of life, and to come to peace with the family and their God before the end, when a cure is no longer possible.

One important problem is that we find very few oncologists attend the palliative care sessions at conferences and so sometimes we are just talking to ourselves. We need to encourage oncologists to attend these sessions in order to help the suffering African patient, who should be the centre of all we do.

You want patients to live longer? They do live longer with palliative care, now scientifically proven [3], because without pain they eat better, sleep better and live longer, with a higher quality of life: “they can live until they die”.

## The solution: greater cooperation?

So this is a plea from a veteran in Africa of more than 30 years, begging that our services can be more realistic and also include those patients who never see a health worker. We need to seek them out in their homes and bring relief to them. If we confine ourselves to only our hospital patients, seeing the illness rather than the person, family and community behind the illness, we are not empathising with the patient. We fail to realise that one day this time will come for all of us. We see cure as a success and dying as a failure. But the failure is lack of care, failing to move with advances in pain and symptom control because of myths, still being taught even now in our medical schools, cautioning the use of this God given remedy for pain.

So let us move together in the next 20 years. AORTIC was founded to help the cancer patients in Africa that suffer so much. AORTIC is in a prime position to bring us together. Let the research and training, highlighted in the name of AORTIC, encourage us to work towards relieving the suffering of patients and families, by meeting their needs, using our science in practical ways to bring peace and comfort at this special time of life.

## Notes

*Access free online accredited *education modules* on palliative care.*

Watch *Prof Merriman discuss these issues on ecancer tv.*
